# Human Chitotriosidase: Catalytic Domain or Carbohydrate Binding Module, Who’s Leading HCHT’s Biological Function

**DOI:** 10.1038/s41598-017-02382-z

**Published:** 2017-06-05

**Authors:** Oscar Crasson, Gaston Courtade, Raphaël R. Léonard, Finn Lillelund Aachmann, François Legrand, Raffaella Parente, Denis Baurain, Moreno Galleni, Morten Sørlie, Marylène Vandevenne

**Affiliations:** 1 InBioS - Center for Protein Engineering, Department of Life Sciences, Université de Liège, Sart-Tilman, B4000 Liège Belgium; 2 Department of Biotechnology, Norwegian Biopolymer Laboratory (NOBIPOL), NTNU - Norwegian University of Science and Technology, N-7491 Trondheim, Norway; 3 InBioS - Eukaryotic Phylogenomics, Department of Life Sciences and PhytoSYSTEMS, Université de Liège, Sart-Tilman, B4000 Liège Belgium; 4 Department of Chemistry, Biotechnology and Food Science, Norwegian University of Life Sciences, N-1432 Ås, Norway

## Abstract

Chitin is an important structural component of numerous fungal pathogens and parasitic nematodes. The human macrophage chitotriosidase (HCHT) is a chitinase that hydrolyses glycosidic bonds between the N-acetyl-D-glucosamine units of this biopolymer. HCHT belongs to the Glycoside Hydrolase (GH) superfamily and contains a well-characterized catalytic domain appended to a chitin-binding domain (ChBD_CHIT1_). Although its precise biological function remains unclear, HCHT has been described to be involved in innate immunity. In this study, the molecular basis for interaction with insoluble chitin as well as with soluble chito-oligosaccharides has been determined. The results suggest a new mechanism as a common binding mode for many Carbohydrate Binding Modules (CBMs). Furthermore, using a phylogenetic approach, we have analysed the modularity of HCHT and investigated the evolutionary paths of its catalytic and chitin binding domains. The phylogenetic analyses indicate that the ChBD_CHIT1_ domain dictates the biological function of HCHT and not its appended catalytic domain. This observation may also be a general feature of GHs. Altogether, our data have led us to postulate and discuss that HCHT acts as an immune catalyser.

## Introduction

Carbohydrate recognition processes are involved in numerous regulatory pathways such as cell signalling and proliferation, fertilization, embryogenesis and in diseases like cancers. Carbohydrates also play a critical role in pathogen recognition, inflammation and innate immune responses through a large array of carbohydrate binding proteins^1^. Chitin, a water-insoluble homopolysaccharide composed of β-1,4-linked N-acetyl-D-glucosamine (GlcNAc) units, is an abundant structural component of arthropods and various infectious organisms like protozoans (e.g. Plasmodium falciparum), nematodes and fungi^2, 3^. As mammals do not produce chitin, this polymer is likely a strategic target for innate immune agents. Defense proteins, including lectins, are known to play a crucial role in the initiation of innate immune mechanisms^4^. These carbohydrate binding proteins include numerous members, which are notably synthetized by many organisms including plants and animals, thereby highlighting their ubiquity and necessity for survival^5^. Some of these lectins are able to bind reversibly to chitin and include a conserved structural motif termed “hevein-fold”; which is rich in polar and aromatic residues^6^. Genes encoding these motifs, associated or not with a catalytic domain, are usually expressed after exposure to chitin-containing pathogens. Among other characteristics, lectins have been shown to bear remarkable anti-fungal properties^7, 8^. The macrophage chitotriosidase (HCHT) is one of the three active chitinases synthetized by humans, together with acidic mammalian chitinase (AMCase) and the recently discovered exochitinase, chitobiase (CTBS)^9–11^. This protein is synthetized as a 50-kDa soluble monomeric enzyme that is able to hydrolyse colloidal chitin. This modular protein is composed of a catalytic domain that belongs to family 18 of glycoside hydrolases (GH18) and of a carbohydrate-binding module, named ChBD_CHIT1_. The latter domain is stabilized by 3 disulphide bonds and was classified in the CAZy (Carbohydrate-Active enZYmes) database (http://www.cazy.org) in family 14 (CBM14). This family includes small binding domains like lectin-like proteins and is characterised by the presence of the highly conserved “hevein-fold”. Among the three human chitinases, HCHT has drawn most of the attention and is nowadays known to be involved in innate immunity for several reasons. Firstly, this enzyme is mostly synthesized by human macrophages that play a critical role in innate immunity^12^. Secondly, HCHT is overexpressed in several pro-inflammatory diseases and in various human sub-populations more exposed to infectious organisms^13, 14^. Finally, the expression of HCHT’s gene can be modulated by the action of cytokines and different immune inducers^15^. However, the precise role of HCHT in innate immunity and its associated molecular mechanisms remain unclear. Indeed, most of the work reported on this enzyme focused on the association of its expression with various diseases. Hence, HCHT is used as a biomarker for the diagnostic of Gaucher disease, sarcoidosis, glucose intolerance, β-thalassemia and World Trade Center lung injury^16–18^.

In bacteria, insoluble chitin hydrolysis requires the synergic activity of several chitinases. For example, Serratia marcescens expresses a battery of chitinases with complementary activities including exochitinases (ChiA and ChiB), endochitinases (ChiC), a N-acetyl-hexosaminidase (chitobiase) and a lytic polysaccharide monooxygenase (CBP21) that act together to hydrolyse efficiently chitin containing structures in order to generate an energy source^19, 20^. In human, in the context of innate immunity, hydrolysis of chitin-containing pathogens would also require complementary chitinase activities. Nevertheless, HCHT appears to be the only chitinase secreted by macrophages. It seems unlikely that HCHT alone could efficiently degrade chitin-containing microorganisms. This intriguing observation explains why the exact function of this enzyme in the context of innate immunity remains unclear.

The aim of this study was to analyse the chitin-binding properties of HCHT chitin binding domain (ChBD_CHIT1_). Our data provide the molecular basis for chitin recognition by ChBD_CHIT1_ and, given the high conservation of the residues involved in chitin binding amongst CBMs 14 as well as other CBM families, we postulated that this binding mode is a hallmark of CBM-carbohydrate interactions.

Moreover, we have interrogated the biological function of HCHT, and more specifically we attempted to understand how such an atypical association between a lectin-like CBM14 (ChBD_CHIT1_) and a glycoside hydrolase (GH18) has been conserved through evolution to generate HCHT homologues involved in defense mechanisms. Our data suggest that ChBD_CHIT1_ has evolved by recruiting a glycoside hydrolase domain, initially used for metabolic purposes in other organisms, to become an important component of innate immunity in humans.

## Results

### Interaction between ChBD_CHIT1-49_ and carbohydrates

**Figure 1:**
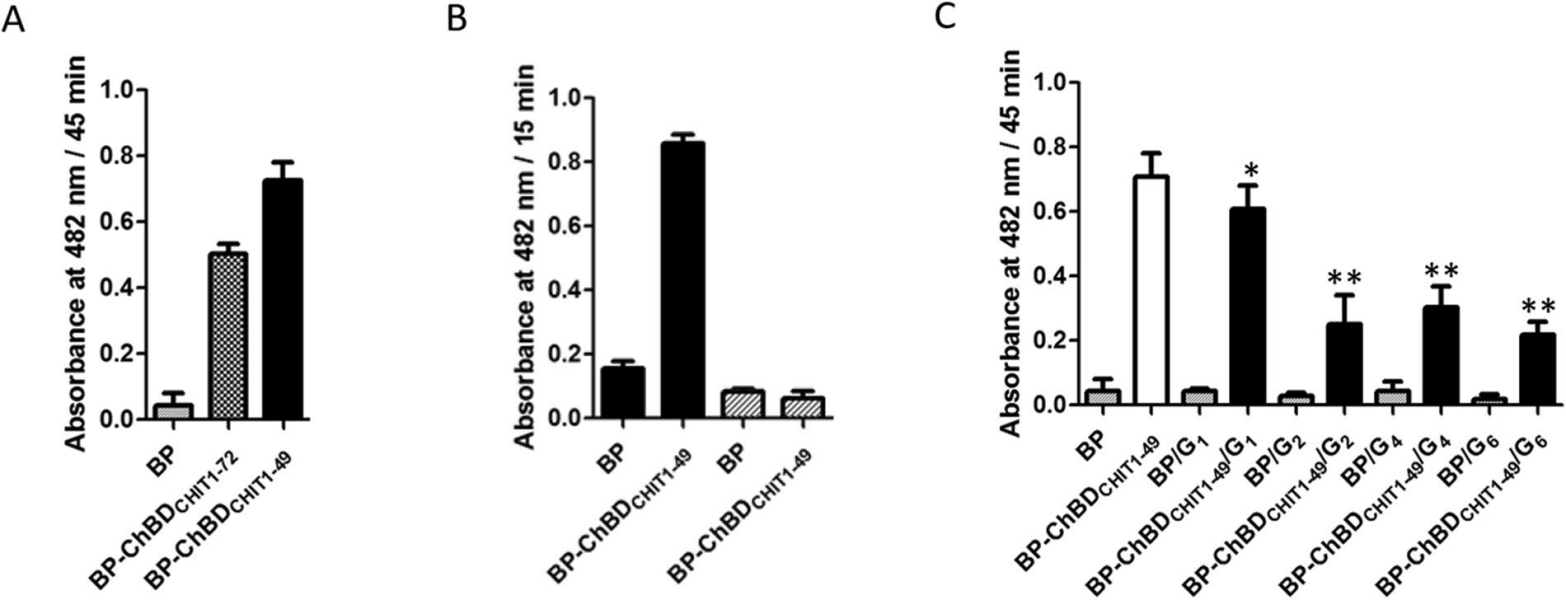
Binding assays of BP-ChBD_CHIT1-49_ towards different ligands. (**A**) Insoluble chitin was first used as a ligand to compare the binding efficiency of the protein used in the present study (BP-ChBD_CHIT1-49_) and the one reported in our previous work^21^ (BP-ChBD_CHIT1-72_) using the reporter enzymatic activity of the carrier protein BlaP. The β-lactamase BlaP without any inserted ChBD_CHIT1_ (labelled BP) was used as a negative control (**B**) Hyaluronan (HA; 50% acetylated; black) and peptidoglycan (PG; 50% acetylated; hatched) were also tested using the same procedure. (**C**) GlcNAc_1_, GlcNAc_2_, GlcNAc_4_ and GlcNAc_6_ (respectively G_1_, G_2_, G_4_ and G_6_) were used as competitors. An equimolar amount of competitor was pre-incubated with the protein before incubating the mixture with insoluble chitin. Except for GlcNAc_1_ (*), all competitors showed a similar inhibition effect (**).

In order to easily monitor the chitin binding activity of ChBD_CHIT1_, we inserted the 49 C-terminal residues of HCHT into the BlaP β-lactamase and the chitin binding activity of the resulting hybrid protein was monitored using the β-lactamase enzymatic activity as a reporter as previously described^21^. Our data indicated that ChBD_CHIT1-49_ displayed chitin-binding activity on chitin-coated magnetic beads (Fig. 1A). Since N-acetyl glucosamine (GlcNAc) is the monomeric subunit of chitin, we have investigated the interaction of ChBD_CHIT1-49_ with different soluble chito-oligosaccharide derivatives (GlcNAc_1_, GlcNAc_2_, GlcNAc_4_ and GlcNAc_6_). In practice, the protein was pre-incubated with a given chito-oligosaccharide (acting as a competitor) and then the mixture was incubated with insoluble chitin before measuring the β-lactamase activity immobilized on insoluble chitin. The data showed a low competition effect of GlcNAc_1_, whereas all the other chito-oligomers showed a significant chitin binding inhibition (Fig. 1C). Notably, the inhibition effect of GlcNAc_2_, GlcNAc_4_ and GlcNAc_6_ were in the same order of magnitude. These data suggest that the interaction surface of ChBD_CHIT1-49_ is relatively small and that the residues involved in binding are localised to a limited area.

Other polysaccharides were also tested and ChBD_CHIT1-49_ was found to bind hyaluronan (50% acetylated). Interestingly, no interaction was detected for Escherichia coli peptidoglycan (Fig. 1B), which also includes GlcNAc units.

### Molecular basis for chitin recognition

**Figure 2:**
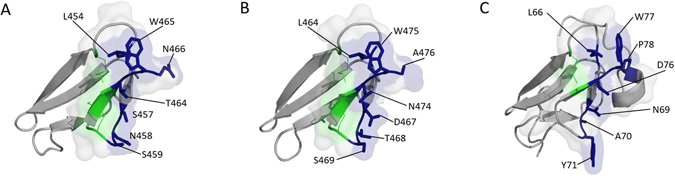
Structures of ChBD_CHIT1-49_, AMCase (3D model) and tachycitin (*“hevein-fold”* motifs are shown in transparent surface representation). (**A**) Crystal structure of ChBD_CHIT1-49_ (PDB ID: 5HBF). The aromatic and polar residues are represented in blue sticks (conserved disulphide bonds are represented as green lines). (**B**) 3D model of AMCase CBM14 (YASARA) built by sequence homology using the ChBD_CHIT1-49_ X-ray 3D structure as template. The corresponding residues shown in Fig. 2A are highlighted in blue sticks. (**C**) NMR structure of tachycitin (PDB ID: 1DQC) where corresponding residues shown in Fig. 2A are highlighted in blue sticks.

The analysis of the recently solved structure of ChBD_CHIT1-49_ (PDB ID: 5HBF; Fig. 2A) has revealed the presence of a specific structural motif that is also found in tachycitin (PDB ID: 1DQC; Fig. 2C) and hevein (PDB ID: 1T0W)^1, 22, 23^. This motif known as the “hevein-fold” is well-conserved within CBM14 family and has been shown to be involved in chitin binding^1^. In ChBD_CHIT1-49_, this “hevein” motif is stabilized by two disulphide bonds (Cys450-Cys463, Cys460-Cys462).

**Figure 3:**
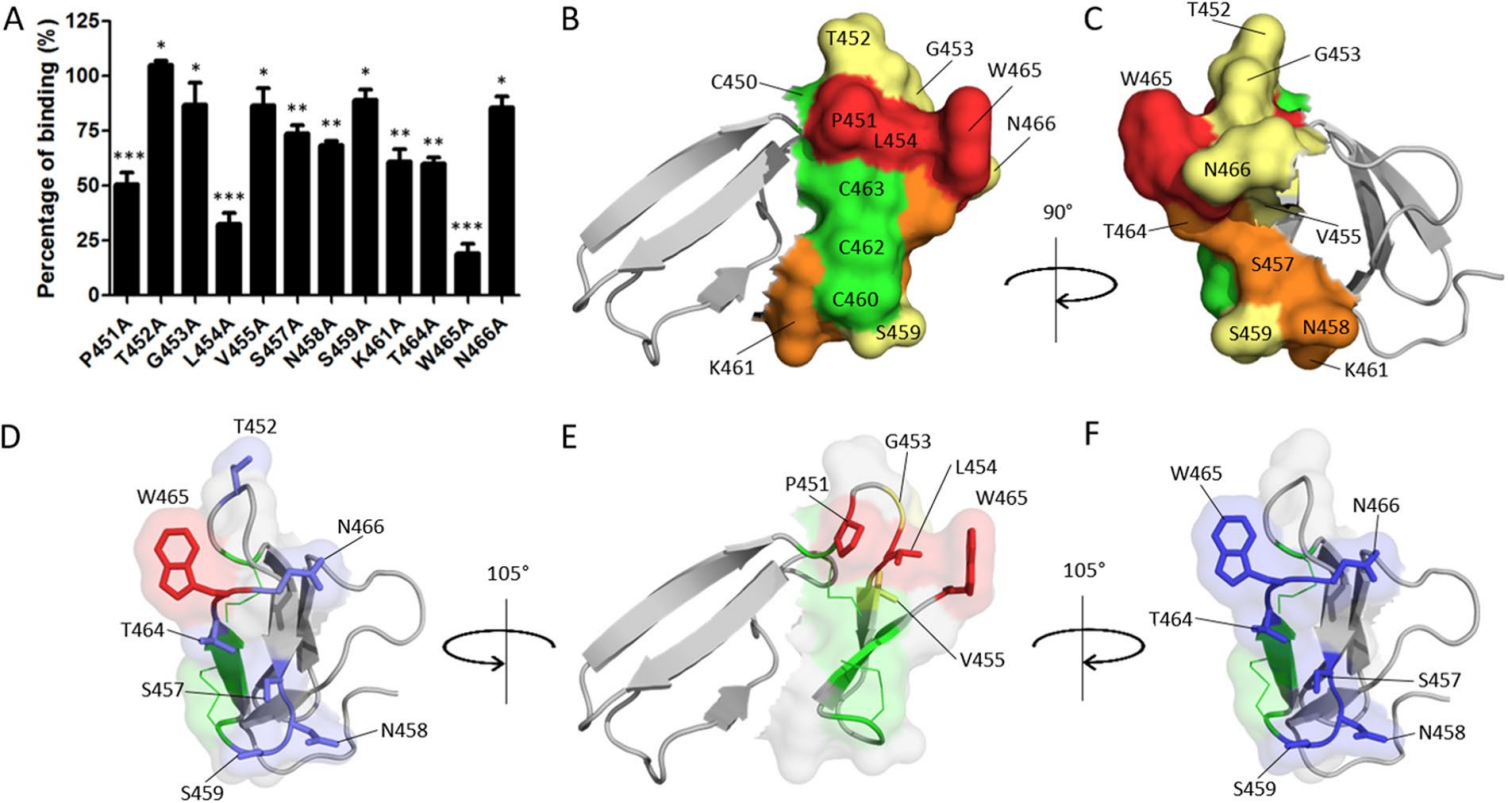
Molecular mechanism of chitin binding and structural features of ChBD_CHIT1-49_. (**A**) The diagram shows the chitin binding efficiencies of single mutants generated by directed mutagenesis of predicted binding residues. All the protein mutants (except T452A) displayed reduced binding activities and were classified in three different groups depending on the impact of the mutation: (i) low impact *, (ii) medium impact ** and (iii) high impact ***. (**B**) X-ray 3D structure of ChBD_CHIT1-49_ solved by Fadel and coworkers (PDB ID: 5HBF) where the *“hevein-fold”* motif is shown in surface representation whereas the rest of the structure is represented in cartoon (disulphide bonds are colored in green). Mutated residues were colored in yellow, orange and red depending respectively on the low, medium and high impact of the mutation on the chitin binding function. (**C**) 90° rotated view of the structure. (**D**) Detailed view of polar residues (in blue sticks) and Trp465 (in red) in the *“hevein-fold”* motif of ChBD_CHIT1-49_ (disulphide bonds in green). (**E**) Representation of key residues (P451, G453, L454 and V455) involved in the hydrophobic pocket which stabilises Trp465 orientation (disulphide bonds in green). (**F**) Surface representation of the binding surface of ChBD_CHIT1-49_ highlighting the key aromatic residue Trp465 and the key polar residues S457, N458, S459, T464 and N458 (blue sticks) directly involved in chitin binding (disulphide bonds in green).

Alanine scanning was used to investigate the “hevein” motif. Each of the 12 residues contained within the “hevein” motif of ChBD_CHIT1-49_ was substituted by alanine using directed mutagenesis. In order to preserve the structural integrity of the protein, cysteines and Phe456 were not mutated. All the mutants were expressed and purified as hybrid β-lactamases in order to use the enzymatic activity of the β-lactamase moiety to monitor the chitin-binding activity of ChBD_CHIT1-49_ mutants. The integrity of the fold as well as the functionality of the corresponding mutated domains were probed using Far-UV CD spectra and enzymatic activity assays, respectively (Figure S1, Table S1). The CD measurements were performed on the isolated ChBD_CHIT1-49_ mutants whereas the β-lactamase enzymatic activity assays were recorded on the corresponding hybrid proteins. In general, the chitin binding affinities of the mutants displayed reduced binding affinity. Mutants were classified into three different groups according to the impact of the mutation on the binding affinity: (i) low impact, Thr452, Gly453, Val455, Ser459 and Asn466; (ii) medium impact, Ser457, Asn458, Lys461 and Thr464; (iii) and high impact, Pro451, Leu454 and Trp465 (Fig. 3A,B,C). The deletion of Trp465 has previously been described to have a deleterious effect on binding^24^, which is in good agreement with our data, since substitution of this residue showed the strongest impact on ChBD_CHIT1-49_ binding activity (Fig. 3A). The π-electrons on the aromatic residue most likely interact with the C-H bond in the pyranose ring of the GlcNAc unit. Besides Trp465, other apolar residues (Pro451, Gly453, Leu454 and Val455), mostly located in the same loop, appear to be important for binding (Fig. 3A). On the ChBD_CHIT1-49_ structure, these residues form a hydrophobic pocket that stabilizes the loop conformation and consequently the orientation of Trp465 side chain (Fig. 3E). Furthermore, substitution of several polar residues (Ser457, Asn458, Ser459, Thr464 and Asn466) has an impact on chitin binding. These residues likely contribute to binding by providing hydrogen bonds with the ligand (Fig. 3A,D,F). Indeed, polar residues are commonly found in the protein-sugar interfaces^25, 26^.

### Interaction of ChBD_CHIT1-49_ with chito-oligosaccharides

**Figure 4:**
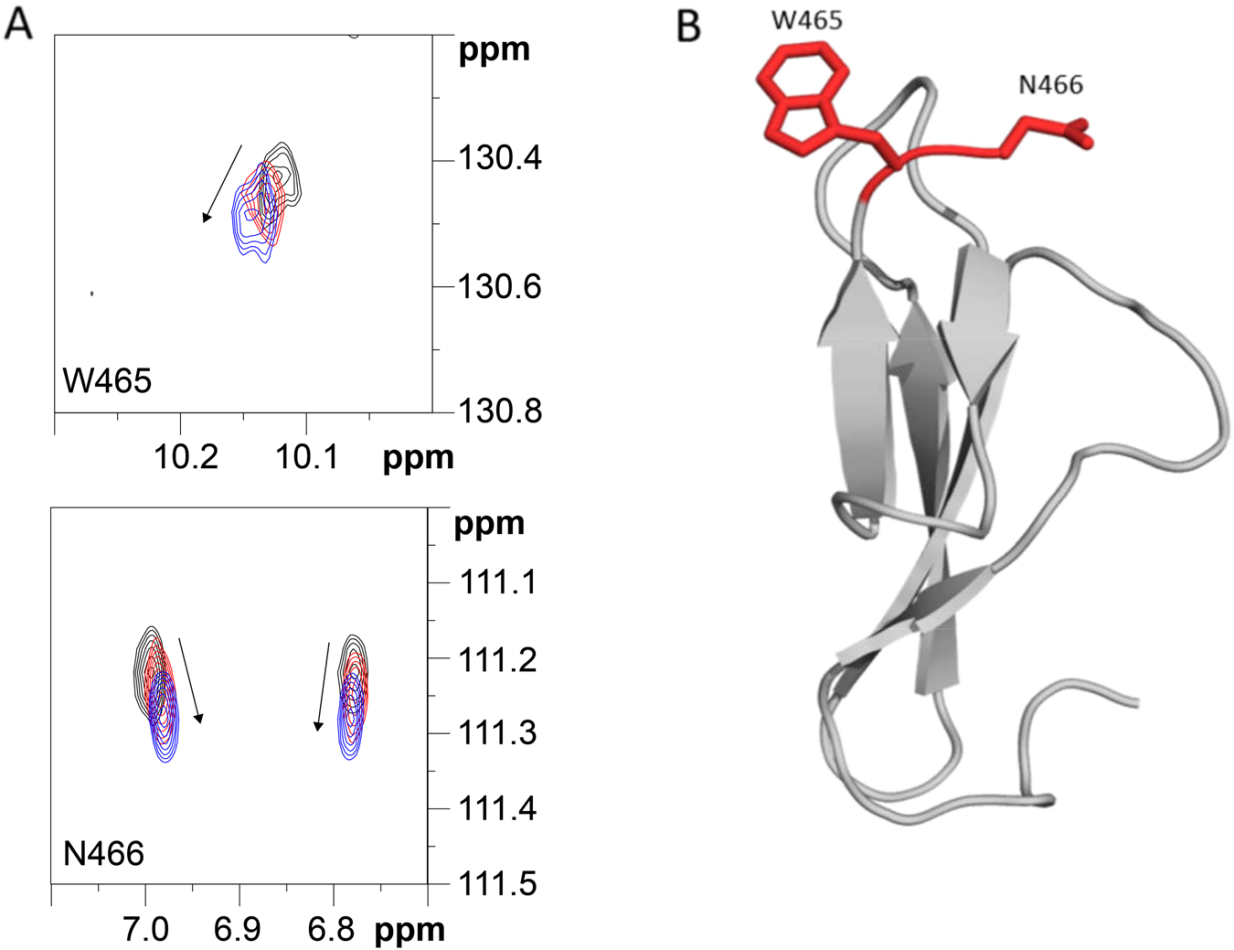
ChBD_CHIT1-49_ interaction with GlcNAc_3_ and binding surface. (**A**) Overlay of an area of interest from the ^15^N-HSQC spectrum for 0.20 mM ChBD_CHIT1-49_ in 50 mM phosphate buffer pH 7.0 recorded at 25 °C (black) in the presence of 4.5 mM (red) and 25 mM GlcNAc_3_ (blue). The arrows indicate direction of the change in chemical shift upon titration. (**B**) The binding surface mapped on the structure of ChBD_CHIT1-49_, where the side chain W465 and N466 are showed in red.

^15^N-HSQC spectra of ChBD_CHIT1-49_ were recorded before and after addition of GlcNAc_3_. The percentages of backbone and side chain assignment covered 92% and 82% of the protein domain, respectively. The side-chain chemical shifts of Asn466 (Hδ1/Nδ1 and Hδ2/Nδ2) and Trp465 (Hε1/Nε1) were the most perturbed in the ^15^N-HSQC spectra upon titration of the ligands. These side-chains, corresponding to the substrate interaction surface, are shown in Fig. 4B. As no other peaks were significantly affected, chemical shift data for these peaks were used to calculate a *K*_*d*_ for GlcNAc_3_ of 9.9 ± 0.8 (SD) mM (Figs 4A and S3B). The measured changes in chemical shifts induced by GlcNAc_2_ were overall too low to calculate a *K*_*d*_ (Figure S3C), however this observation in itself shows that ChBD_CHIT1-49_ binds GlcNAc_3_ stronger than GlcNAc_2_.

### Phylogenetic Study. ChBD_CHIT1-49_ is part of the CBM14 family

**Figure 5:**
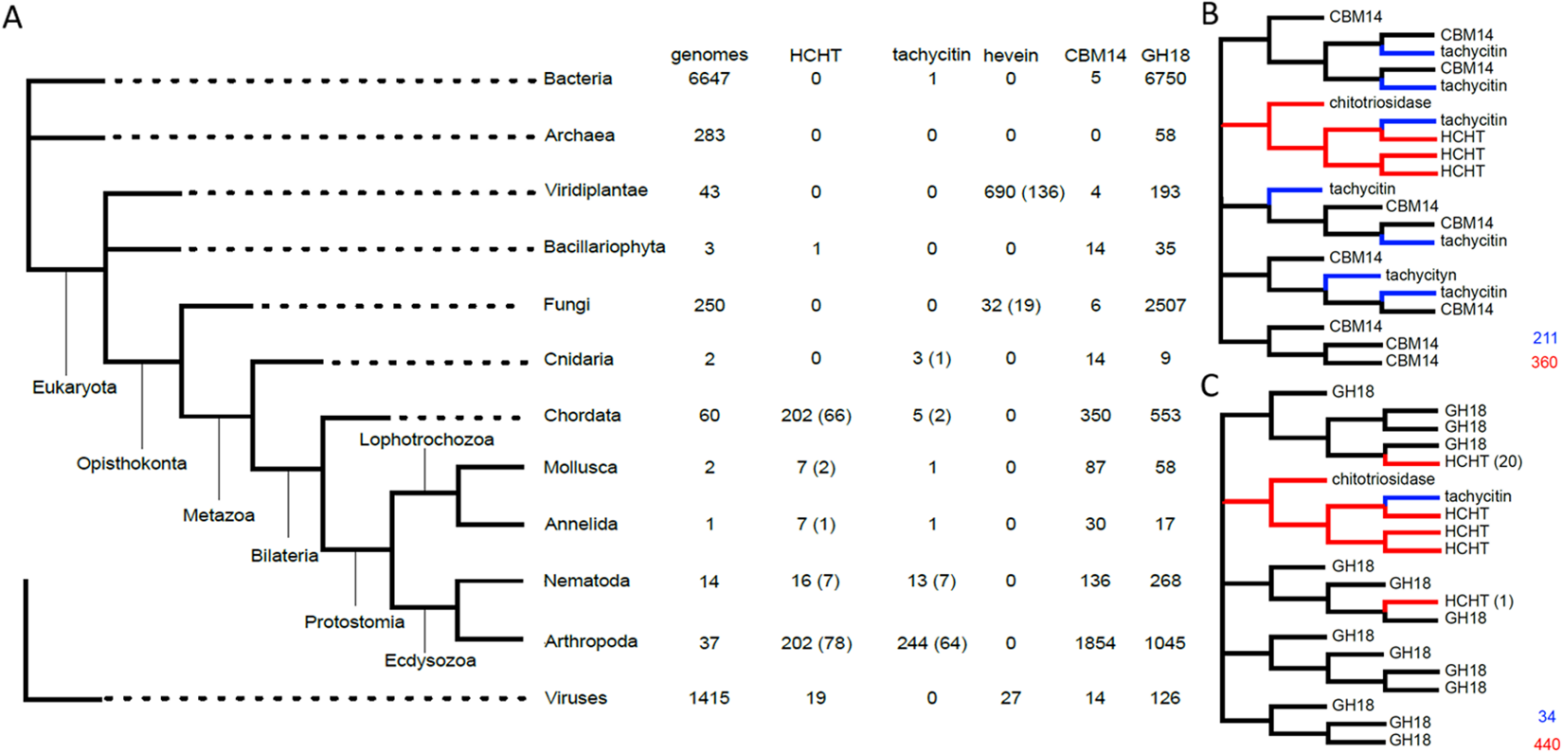
Phylogenetic analyses. (**A**) Reference phylogenetic tree on which are mapped the results of genome mining for chitotriosidase (HCHT-like), tachycitin and hevein proteins, as well as GH18 and CBM14 domains. The number of representative species are written in brackets. (**B**) Schematic tree of CBM14 domains, in which families integrated in HCHT-like architectures are colored in red (families tagged tachycitin are colored in blue). (**C**) Schematic tree of GH18 domains, in which families integrated in HCHT-like architectures are colored in red (families tagged tachycitin shown in blue).

In HCHT, this CBM is associated by a linker region to a catalytic domain belonging to the GH18 family. Although phylogenetic studies have previously been reported on the GH18 family^27^ and CBMs14^28^, we have analyzed the taxonomic distribution of CBMs 14, GHs 18 and HCHT in order to trace back the possible origin and evolution of these domains/proteins (Fig. 5A). Hence, we have noticed that GHs 18 proteins are present in genomes of all three domains of Life (Archaea, Bacteria, Eukaryota) and of some viruses. In contrast, CBM14 domains are restricted to specific groups of Eukaryota, suggesting that the appearance of this domain family is more recent. While both domain families (GH18 and CBM14) have coexisted since early eukaryotes [as deduced from their occurrence in both unikont and bikont lineages]^29^, HCHT-like proteins apparently only assembled in the ancestor of bilaterian animals (i.e., after the divergence of sponges, ctenophores and cnidarians; Fig. 5A). Next we analysed the origin of “hevein-fold” containing proteins, including tachycitin and hevein itself. Tachycitin is also a CBM14 that shares 34% amino acid sequence identity and structure conservation with ChBD_CHIT1-49_. In contrast, hevein, the first protein in which the “hevein-fold” has been described and characterized, is related to the CBM18 family and is strictly present in Viridiplantae, Fungi and viruses genomes (Fig. 5A).

Therefore, our data support the fact that the structure similarity between the “hevein-folds” of CBMs 14 and CBMs 18 appears to be the result of convergent evolution, which is commonly admitted by the scientific community^23^. Finally, we built domain trees for CBM14 and GH18 families. The CBM14 tree features a subtree that includes all CBMs 14 involved in HCHT-like architectures (Fig. 5B), whereas, in the GH18 tree, most (but not all) of the GHs 18 involved in HCHT-like architectures are located in the same subtree (Fig. 5C). Considering the weak resolution of these two domain trees, it is very possible that the additional HCHT-like GH18 domains that are not part of the main HCHT-like subtree are simply misplaced by phylogenetic inference. This would mean that HCHT-like proteins are all homologous because they have a single origin, tracing back to the original association of a CBM14 domain and of a GH18 domain in a common ancestor of Bilaterians.

## Discussion

Our work focuses on the CBM of the HCHT and has highlighted several features of this domain that might be applied to many other CBMs. First, we have identified the chitin binding residues of ChBD_CHIT1-49_. We have also characterized and quantified the interaction between ChBD_CHIT1-49_ and small chito-oligosaccharides using NMR spectroscopy and competition binding assays. Our data indicate that although ChBD_CHIT1-49_ interacts tightly to insoluble chitin, the measured affinities for chito-oligosaccharides are weak with dissociation constant values in the mM range. A phylogenetic study was also conducted to analyze the evolutionary paths of both domains included in HCHT (CBM14 and GH18) in order to determine when these domains were combined to give the GH18-CBM14 architecture found in HCHT and how this association is related to the biological role of this chitinase in innate immunity.

### A hydrophobic pocket present on CHBD_CHIT1-49_ “hevein” motif important for chitin binding

Our alanine scanning experiment has highlighted the presence of a hydrophobic pocket that includes Leu454 and Val455, both located in a loop induced by Pro451. We hypothesized that this hydrophobic pocket orients the Trp465 indole ring (Fig. 3E) for chitin binding. Molecular dynamic simulations (YASARA) performed on the Leu454Ala mutant; which presented the most impaired chitin binding activity, support this hypothesis. Indeed, the Trp465 indole side chain of the generated mutant exhibits higher flexibility and display more conformational freedom (Figure S2). These observations suggest that Leu454 plays an important role in orientating Trp465 side chain. Also, Pro451, Gly453, Leu454 and Trp465 are relatively well conserved through CBMs 14, which suggests that this binding mechanism can be generalizable to other CBMs 14 (Fig. 2). Our alanine scanning experiment has also highlighted that mutation of five polar residues (Ser457, Asn458, Ser459, Thr464 and Asn466) decreased the chitin binding efficiency. These residues are all located below the Trp465 side chain and well positioned to form hydrogen bonds (Fig. 3D,F). We postulated a chitin binding mechanism for ChBD_CHIT1-49_ where Trp465 oriented by its hydrophobic pocket recognizes and interacts with a first GlcNAc unit of the polysaccharide. Besides this main interaction, polar residues located below Trp465 seem perfectly positioned to interact with a second and probably a third GlcNAc unit. This molecular mechanism reflects very well the binding mechanism of Type C CBMs, where essentially polar residues located in loops and few aromatic residues are directly involved in chitin recognition^6^.

### The chitin-binding surface of ChBD_CHIT1-49_ can efficiently fit a minimum of two GlcNAc units

Our competition binding assays presented in Fig. 1C has highlighted important information regarding the chitin binding surface of ChBD_CHIT1-49_. These data showed that the smallest chito-oligosaccharide able to compete with insoluble chitin is GlcNAc_2_. Importantly, GlcNAc_1_ showed a much lower inhibition effect compared to GlcNAc_2_. This suggests that at least two GlcNAc units are required to form a stable complex. Moreover, NMR titration showed that a third GlcNAc unit could extend the surface area of the interaction (Figs 4A and S3). Notably, these data contrast with our previous study that didn’t show any chitin-binding inhibition of ChBD_CHIT1_ by the chito-oligomers^30^. In the present study, we used another type of chitin (chitin-coated magnetic beads) rather than α-crystalline chitin in our previous work. In addition, the amount of chitin that we used herein was much lower compared to our previous study, which placed us in better condition to see a chitin binding inhibition by chito-oligosccharides given their low binding affinity (*K*_*ds*_ in the mM range) for ChBD_CHIT1_.

It is also interesting to comment on the difference in the binding affinities of ChBD_CHIT1-49_ towards soluble chito-oligosaccharides and crystalline chitin. Indeed, although it is difficult to quantify crystalline chitin interaction given the insoluble character of this ligand, we estimated that this interaction is tight given the harsh and denaturing conditions required to remove ChBD_CHIT1-49_ from a crystalline chitin support. In contrast, the measured *K*_*ds*_ for chito-oligosaccharides are in the high mM range, which is weak (Figs 4A and S3B). In the context of chitin hydrolysis by HCHT, the weak affinity for small chito-oligosaccharides, which are the hydrolysis products of the enzymatic reaction, is an advantageous feature that avoids ChBD_CHIT1-49_’s binding inhibition, which maintains HCHT efficient towards crystalline chitin.

### New insights into the modularity of HCHT using a phylogenetic study approach

HCHT is a modular enzyme composed of a GH18 (catalytic domain) and a CBM14 and is known to be involved in innate immunity by indirect evidences (see Introduction). In this work, we have performed a phylogenetic study (Fig. 5) on the catalytic and chitin binding domains of HCHT. The results showed that GH18-containing genes are found in genomes of all domains of life and that this ancestral family of catalytic domains is present in numerous modular enzymes involved in various biological processes^20, 31–33^. It is therefore not surprising that they are associated to a wide variety of domains as it is illustrated by the 377 different architectures classified in Pfam database (http://pfam.xfam.org). In contrast, CBMs 14 are only found in Eukaryota (Fig. 5A), which implies that this protein family is related to strictly eukaryotic biological functions like innate immunity. Interestingly, according to Pfam database CBMs 14 are mostly associated with domains involved in defense/immune functions.

Conservation of the HCHT-like architecture only occurred from Bilateria (Fig. 5A). This lineage includes animals with higher complexity and presumably, more developed immune systems. In these bilaterians, HCHT-like proteins were described as a component of defense and development^34^. All these observations led us to an unexpected conclusion: the leader domain that dictates the biological function and dominates the biological activity of HCHT might not be the catalytic domain but rather the CBM14. If this hypothesis is confirmed, it will be of crucial importance because it would allow us to better understand and predict the biological role of CBM-containing proteins.

## Conclusion

Altogether, the data presented in this work bring new insights into the biological function of HCHT. Although there are several evidences that link HCHT to immunity, the precise role of this chitinase in human remains unclear. It is reasonable to postulate that the presence of ChBD_CHIT1-49_ facilitates the recognition of small and hardly accessible motifs in different chitin types. These features probably enhance HCHT’s capability to be efficient towards a huge diversity of chitin containing pathogens. Since chito-oligosaccharides were recently shown to display a higher immunogenicity compared to insoluble chitin^35^, the lectin-like behaviour of ChBD_CHIT1-49_ might confer to HCHT the ability to enhance the inflammatory response against a wide variety of chitin containing pathogens by releasing immunogenic chito-oligosaccharides and therefore acting as an immune catalyser that can lead to a recruitment of additional immune actors.

## Methods

### Construction of the Genes Encoding the Hybrid β-lactamases

The genes encoding the chitin-binding domains ChBD_CHIT1-72_ (residues Pro395 to Asn466) and ChBD_CHIT1-49_ (residues Thr418 to Asn466) of the HCHT (Uniprot number: Q13231) were amplified by PCR and inserted into the gene coding for the class A β-lactamase exo-small BlaP (BP)^36^ previously cloned in the expression vector pET26b(+). The insertion site is located between residues Asp197 and Lys198 of the β-lactamase and the insertions of the gene fragments into the BlaP gene were performed as described in our previous studies^21, 30, 37, 38^. It is important to note that, in our previous work, we used a slightly longer ChBD_CHIT1_ that included the 72 C-terminal residues of HCHT (residues Pro395 to Asn466), however based on sequence alignments; we noticed that only the 49 C-terminal residues of ChBD_CHIT1_ were conserved. In addition previous studies published by Tjoelker and coworkers^24^ as well as Fadel and coworkers^22^ confirmed that the minimum length chitin-binding domain is ChBD_CHIT1-49_. This is why we shortened the domain down to 49 residues. Furthermore, the inserted ChBD_CHIT1-49_ gene was surrounded by two thrombin cleavage sites to release the isolated ChBD_CHIT1_ domain after production and purification when needed as reported previously^21^. The resulting genetic construct, called pET26b(+)-BP-ChBD_CHIT1-49_, was used to express the hybrid β-lactamases harbouring both the pel B signal peptide for periplasmic secretion and a His6 tag sequence at the N-terminal and C-terminal extremities, respectively.

### Alanine Scanning Mutagenesis

We selected a subset of 12 residues present on the predicted chitin-binding surface of ChBD_CHIT1-49_ for substitution into alanine. The 12 single mutants of the protein BP-ChBD_CHIT1-49_ were generated using the Quick-Change Multi Site-Directed Mutagenesis kit (Agilent Technologies) following the manufacturer’s instructions. Briefly, mixtures of 50% Phusion PCR Master Mix (Thermo Fisher Scientific), 5% DMSO, 100 ng of template DNA and 2 mM of primers were submitted to the following PCR program: 30 s at 98 °C, 10 s at 98 °C, 30 s at 42 °C and 15 s at 72 °C (30 cycles), followed by a final step at 72 °C for 10 min. The PCR products were then digested by DpnI in FastDigest Buffer (Thermo Fisher Scientific) for 10 min at 37 °C to remove template DNA. Finally 10 μL of the resulting mutated plasmids were used to transform E. coli DH5α competent cells for plasmid amplification.

### Hybrid Protein Expression and Purification

All hybrid proteins were expressed in E. coli BL21(DE3). Transformed cells were cultured at 37 °C in Terrific Broth medium supplemented with 50 μg/mL kanamycin until OD600nm reached 2.5. Cultures temperature were then decreased to 18 °C for 30 min before proceeding to the induction of protein expression by the addition of isopropyl β-thiogalactopyranoside (IPTG) to a final concentration of 1 mM. Cultures were grown for 14 h at 18 °C. Cells were harvested by centrifugation (twice) and resuspended successively in two distinct periplasmic extraction buffers: firstly in 1/10th volume of 20 mM Tris 600 mM sucrose 5 mM EDTA pH 8.0 at 37 °C and secondly in 1/4th volume of 5 mM MgSO_4_ at 4 °C. Each periplasmic extract was supplemented with one tablet of complete Protease Inhibitor Cocktail (Roche).

Periplasmic proteins were loaded on a 5 mL Bio-ScaleTM Mini ProfinityTM IMAC column (BIO-RAD) equilibrated in 300 mM KCl, 50 mM KH_2_PO_4_ and 5 mM imidazol pH 8.0. The column was successively washed with 300 mM KCl, 50 mM KH_2_PO_4_, 10 mM imidazol pH 8.0 and the elution was performed with 300 mM KCl, 50 mM KH_2_PO_4_ and 250 mM imidazol pH 8.0. Imidazol was immediately removed using a 50 ml Bio-ScaleTM Mini Bio-gel® P-6 Desalting column (BIO-RAD). Protein purity level and homogeneity were confirmed by SDS-PAGE and UV-Visible (125–400 nm) spectra. Protein concentrations were determined by UV absorbance at 280 nm.

Isolated native and mutated ChBD_CHIT1-49_ were released from their carrier protein BlaP by thrombin cleavage as previously described^21^. In this study, isolated ChBD_CHIT1-49_ domains were purified by molecular exclusion chromatography using XK 26/100 SuperDex 75 PrepGrade column (GE Healthcare) equilibrated in 150 mM NaCl 50 mM phosphate buffer pH 7.5 (PBS).

### Isotopic Labelled Protein Expression and Production for NMR studies

^13^C, ^15^N or ^15^N ChBD_CHIT1-49_ samples were expressed in *E. coli* BL21(DE3) cells. Pre-culture were grown in LB medium (10 g/L tryptone, 5 g/L yeast extract and 5 g/L NaCl) supplemented with 50 μg/mL kanamycin in a shaking incubator at 225 rpm, 30 °C overnight. A 2L LB main culture with 50 μg/mL kanamycin was inoculated with 1% of the overnight culture and grown in a shaking incubator at 225 rpm, 30 °C to OD600nm reaches ∼0.8. Cultures were centrifuged at 4,500 g for 10 min and resuspended on ice in 500 mL M9 media (6 g/L Na_2_HPO_4_, 3 g/L KH_2_PO_4_, 0.5 g/L NaCl) supplemented with 99% (^15^NH_4_)2SO_4_, 98% ^13^C_6_-D-glucose, 10 mL Bioexpress Cell Growth Media (Cambridge Isotope Laboratories, Tewksbury, MA, USA), 10 mL Gibco™ MEM Vitamin Solution (100x), 1 mL 1 M MgSO_4_, 10 mL Trace Metal solution (0.1 g/L ZnSO_4_, 0.8 g/L MnSO_4_, 0.5 g/L FeSO_4_, 0.1 g/L CuSO_4_, 1 g/L CaCl_2_) and 50 μg/mL kanamycin. Expression was induced 15 min after the media change by IPTG to a final concentration of 0.1 mM, and then the culture was incubated at 16 °C, 225 rpm for 20 hours. The cells were harvested by centrifugation at 5,000 g, 10 min, 4 °C and suspended in 30 mL TES buffer pH 8.0 (3.63 g/L TRIS, 1.86 g/L EDTA, 200 g/L sucrose) together with half a tablet Complete Protease Inhibitor (Roche) followed by a centrifugation at 10 min, 4 °C, 6,150 g. The supernatant was removed and the cells incubated at room temperature for 10 min before being resuspended in 25 mL MQ and half a tablet Complete Protease Inhibitor (Roche). The suspension was supplemented with 125 μL 1 M MgSO_4_ before the final centrifugation at 13,000 g, 45 minutes. The supernatant was filtered through a 0.22 μm Sterile-flip filter unit from Nalgene. Labelled ChBD_CHIT1-49_ was purified as described above.

### Enzymatic Characterization of the Hybrid Proteins

The kinetic parameters of the purified hybrid β-lactamases were determined by measuring the rates of nitrocefin (CalBiochem) hydrolysis at different substrate concentrations. Initial rates were measured so that less than 10% of substrate was hydrolysed. A protein concentration of 25 ng/mL was used in the presence of 0.1 mg/mL BSA (Fermentas) used as a crowding and stabilizing agent. The experiment was performed at 37 °C in PBS (pH 7.5). A spectrophotometer (PowerWave X, TempLab) was used to monitor the formation of the hydrolysis product at 482 nm. Kinetic parameters (kcat, Km and kcat/Km) were determined for each hybrid protein as described by Matagne and coworkers^39^. Standard deviations were calculated on the basis of the results obtained from 3 technical replicates for each hybrid protein.

#### NMR Spectroscopy

NMR spectra of 0.2 mM ChBD_CHIT1-49_ samples in 50 mM Phosphate buffer at pH 5.5 and 7.0 were recorded at 25 °C on a Bruker Ascend 800 MHz spectrometer Avance III HD equipped with a 5-mm Z-gradient CP-TCI (H/C/N) cryogenic probe at the NT-NMR-Center/Norwegian NMR Platform (NNP). NMR data were processed using Bruker TopSpin version 3.5. NMR spectral analysis was performed using CARA version 1.5.5^40^. A partial backbone assignment was accomplished using HNCA, CBCA(CO)NH, HN(CA)CO, HNCO, ^15^N-HSQC-NOESY and ^15^N-HSQC spectra.

NMR titration was used to probe the interaction of ChBD_CHIT1-49_ with its ligands chitotriose (GlcNAc_3_) and chitobiose (GlcNAc_2_). For GlcNAc_3_, the titration points were 1.0 mM, 2.4 mM, 4.5 mM, 6.3 mM, 11.6 mM, 17.1 mM and 25.0 mM. For GlcNAc2, the titration points were 0.5 mM, 1.0 mM, 2.4 mM, 4.5 mM, 8.7 mM, 12.4 mM, 18.7 mM and 23.9 mM. 1D-proton and ^15^N-HSQC spectra (at 4096 × 1024 point resolution) were recorded for each titration point.

The side-chains of Trp465 and Asn466 were identified based on the partial assignment and their ^15^N-HSQC peaks were used as reporters for the interaction by measuring chemical shift changes in the N and H^N^ atoms of the backbone of ChBD_CHIT1-49_. A compound change in chemical shift, Δδ*comp* (in ppm) was calculated using the following formula: Δδ*comp* = [(Δδ_*H*_)^2^ + (Δδ_*N*_/x)^2^]^1/2^. Δδ*H* is the change in chemical shift of the amide proton (ppm), Δδ*N* is the change in chemical shift of the amide nitrogen (ppm), and x is a constant used to achieve equal contributions from changes in N and H^N^ shifts, which was set to 6.5^41^.

Equation (1)^42^ was used to estimate the dissociation constant (*K*_*d*_) of the interaction by using Excel to simultaneously fit *K*_*d*_ and Q*max* (Δδ*comp* at saturation) for Δδ*comp* at each ligand concentration, [L] (mM), and the protein concentration, [P] (mM), remained constant at 0.2 mM (Fig. S3B).

1 $${\rm{\Delta }}{\rm{\delta }}comp={\rm{Q}}{\max }\frac{[{\rm{P}}]+[{\rm{L}}]+{K}_{d}\,\pm \,\sqrt{{([{\rm{P}}]+[{\rm{L}}]+{K}_{d})}^{2}-4\,[{\rm{P}}]\,[{\rm{L}}]}}{2\,[{\rm{P}}]}$$

### Alanine Scanning Mutagenesis of the chitin-binding surface of ChBD_CHIT1_

Residues expected to interact with chitin were substituted with alanine. To identify critical amino acids, an excess of purified hybrid proteins expressing a mutated ChBD_CHIT1-49_ domain were mixed with a final chitin magnetic beads concentration of 2% (v/v; New England BioLabs) and 0.1 mg/mL BSA. A control was conducted with the carrier protein BlaP without any inserted ChBD_CHIT1_. Binding assays were performed at room temperature by orbital mixing of the protein-chitin beads suspension during 30 min. Bound proteins were harvested by magnetic attraction and washed three times with 500 mM NaCl 20 mM Tris-HCl 1 mM EDTA 0.1% Tween (pH 5.0). Immobilized protein levels on chitin beads were determined by incubation of the beads with nitrocefin and monitoring of antibiotic hydrolysis over time at 482 nm (RT).

### Phylogenetic Analysis

Starting with the sequences of human chitotriosidase (2201442 A), Tachypleus tridentatus (Arthropoda) tachycitin (1DQC_A), and Hevea brasiliensis (Viridiplantae) hevein (AAO63573.1), and using an E-value threshold of 1e^−5^, three separate PHMMER searches were carried out for genome mining on the UniProtKB sequence database, through the HHMI Janelia web portal (http://hmmer.janelia.org/; now available at http://www.ebi.ac.uk/Tools/hmmer/).

For the HCHT search, only the hit sequences simultaneously featuring a CBM14 domain and a GH18 domain were downloaded (both in non-aligned and aligned format). For the two other proteins, all hit sequences were downloaded in non-aligned format. A combination of batch identifier mapping through the UniProt web portal (http://www.uniprot.org/uploadlists/) and custom Perl scripts (Bio-MUST-Core, D. Baurain, R. R. Léonard, unpublished) was then used to recover the complete taxonomic lineage of each sequence. The three non-aligned sequence files were aligned using MAFFT^43^ and the resulting alignments cleared of partial sequences, defined as lacking more than 50% positions of the longest sequence in each alignment (Bio-MUST-Core). Final alignments were then submitted to phylogenetic inference using either RAxML^44^ and the PROTGAMMALGF model^45, 46^ or PhyloBayes^47^ and the CATGTRG model^48, 49^ to produce the trees. Both models yielded largely unresolved but broadly similar trees.

Based on the downloaded HMMER-aligned HCHT file, two additional alignments corresponding to each one of the two domains were generated (Bio-MUST-Core) and used to build two HMM profiles with hmmbuild^50^. These profiles were then pasted on the HHMI Janelia web portal to carry out two separate HMM searches on UniProtKB, using an E-value threshold of 1e^−5^. All hit sequences featuring at least one copy of the corresponding domain were downloaded in non-aligned format and further processed as above (MAFFT, Bio-MUST-Core, RAxML/PhyloBayes) to produce two phylogenetic trees of the CMB14 and GH18 domain families. To locate chitotriosidase, tachycitin and hevein sequences in the domains trees, a semi-automated annotation pipeline was developed so as to highlight the leaves corresponding to sequences recovered in the three initial PHMMER searches.

## Electronic supplementary material

Supplementary informations
