# Supplementary material for: Human Chitotriosidase: Catalytic Domain or Carbohydrate Binding Module, Who’s Leading HCHT’s Biological Function
Source: Sci Rep. 2017 Jun 5;7:2768. doi: 10.1038/s41598-017-02382-z (PMC5459812; doi:10.1038/s41598-017-02382-z)
Supplement: Supplementary file 1 — Supplementary informations [file 41598_2017_2382_MOESM1_ESM.pdf]

Human Chitotriosidase: Catalytic Domain or Carbohydrate Binding Module, Who's Leading HCHT's Biological Function.

Oscar Crasson[1], Gaston Courtade[2], Raphaël R. Léonard[3], Finn Lillelund Aachmann[2], François Legrand[1], Raffaella Parente[1], Denis Baurain[3], Moreno Galleni[1,] Morten Sørlie[4], and Marylène Vandevenne[1*]

Supplementary Information

FIGURE S1

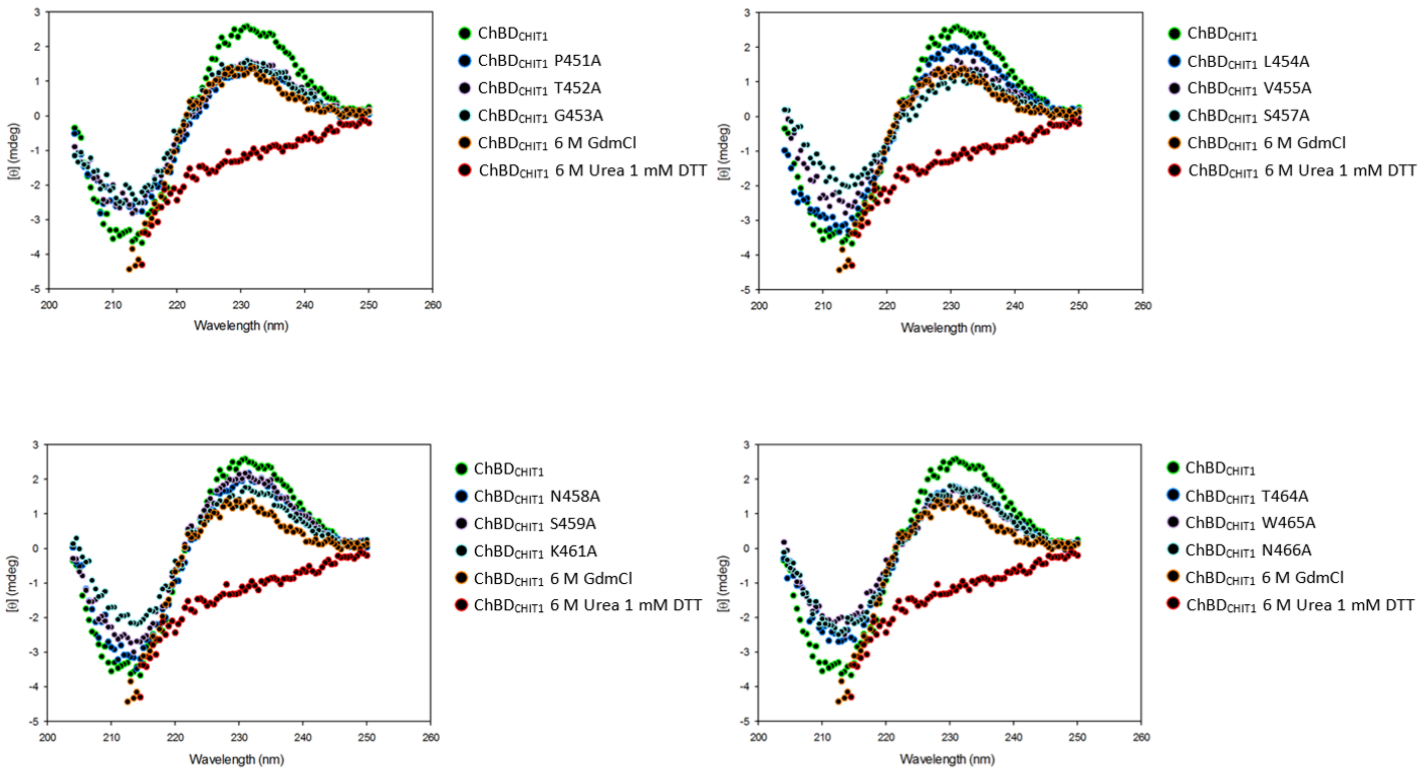

Figure S1. Far-UV CD spectra of ChBDCHIT1-49 and its single mutants. The non mutated ChBD$_{CHIT1-49}$ is represented in green. Unfolded ChBD$_{CHIT1-49}$ samples obtained by incubation with 6 M guanidinium chloride (GdmCl) and 6 M Urea (plus 1 mM DTT) are represented in orange and red dots, respectively. In each spectrum, single mutants are shown in blue, purple and cyan dots. All proteins displayed a β-sheet profile demonstrating that all proteins were well folded and characterized by the expected secondary structure content. Noticeably, the "hevein-fold" motif is a very stable structure that required the addition of reducing agent to abolish the secondary structure content of ChBD$_{CHIT1-49}$ underlining the importance of the disulphide bonds.

FIGURE S2

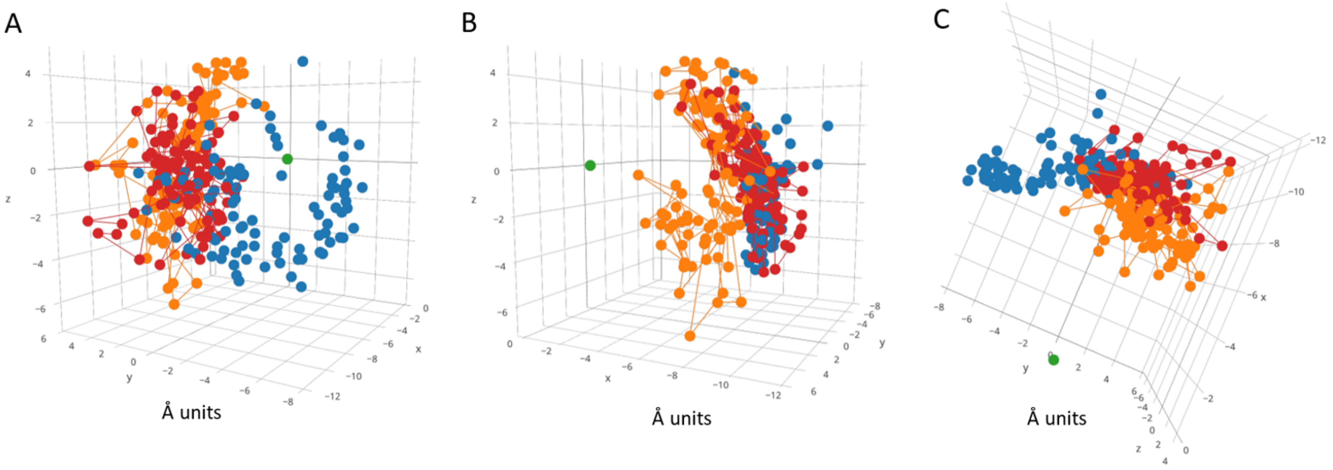

Figure S2. Molecular dynamic simulation (YASARA) of WT ChBD$_{CHIT1\text{-}49}$, Leu454Ala and Leu454Val mutants. The center of mass (COM) of the conserved disulphide bond (Cys450-Cys463; green dot) and the COM of Trp465 have been analysed during a molecular dynamic simulation of 25 ns (100 frames) for the WT ChBD$_{CHIT1\text{-}49}$ (in blue) and the two mutants Leu454Ala (in orange) and Leu454Val (in dark red). In the WT protein, Trp465 COM displayed a well-defined plane with relatively low flexibility due to the steric hindrances and hydrophobic environment of Leu454. For the two mutants, the Trp465 COM is delocalized in the right inner part of the “hevein-fold” and exhibits more flexibility compared to the WT protein, which results in a loss of binding efficiency. (A) Front view. (B) Right view. (C) Top view.

FIGURE S3

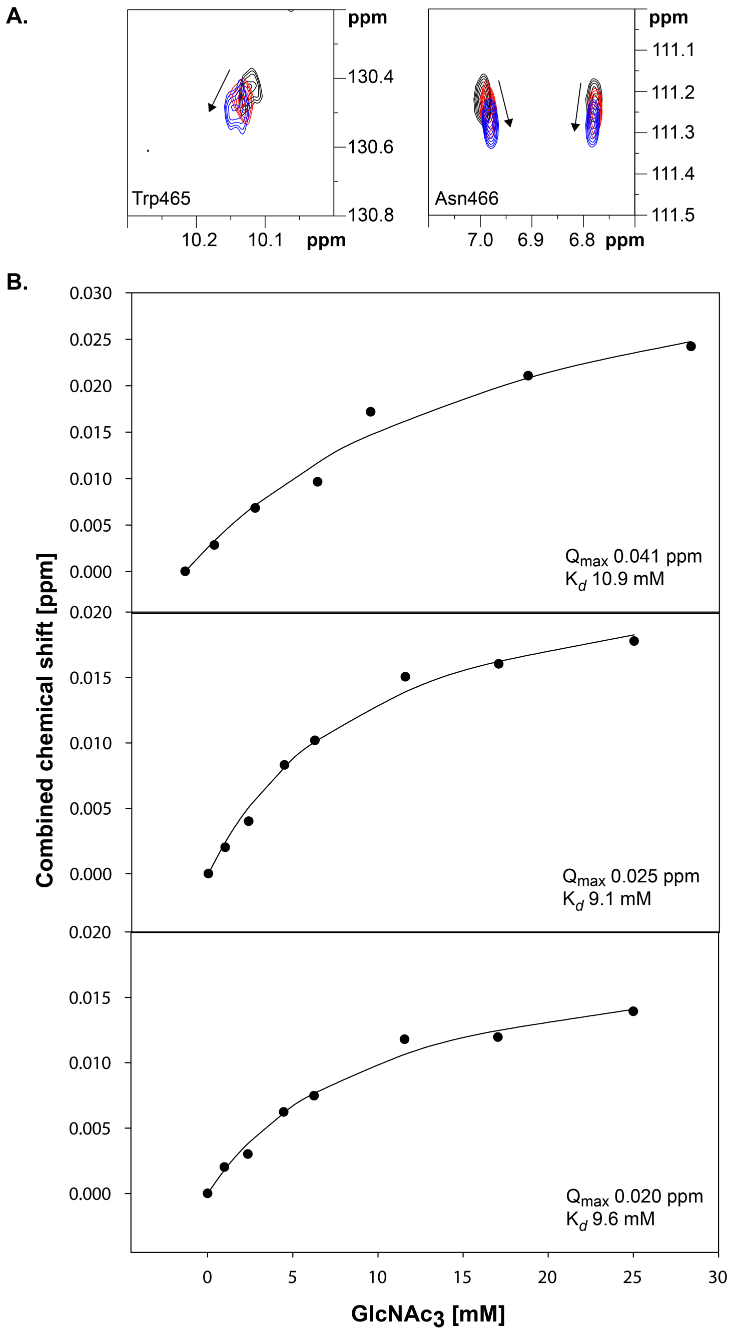

Figure S3. ChBD$_{CHIT1\text{-}49}$ interaction with GlcNAc oligomers. (A) Overlay of an area of interest from the ^{15}N-HSQC spectrum for 0.20 mM ChBD$_{CHIT1\text{-}49}$ in 50 mM phosphate buffer pH 7.0 recorded at 25 °C (black) in the presence of 4.5 mM (red) and 23.9 mM GlcNAc$_2$ (blue). The arrows indicate direction of the change in chemical shift upon titration. (B) Combined chemical shift for three atom pairs [$H^{\varepsilon 1}/N^{\varepsilon 1}$ of Trp465 (top panel) and $H^{\delta 1}/N^{\delta 1}$; $H^{\delta 2}/N^{\delta 2}$ of Asn466 (two lower panels)] upon titration with GlcNAc$_3$. Dissociation constant (K_d) was estimated for individual atom pairs by simultaneously fit K_d and Qmax (Δδcomp at full saturation, and it is assumed equivalent to the bound fraction of the substrate). The average Kd was calculated to 9.9±0.8 (SD) mM for GlcNAc$_3$.

Table S1. Kinetic parameters of BlaP and its chimeric derivatives for nitrocefin

Table S1. *Kinetic parameters of BlaP and its chimeric derivatives for nitrocefin*

Proteins	Km (μM)	k_{cat} (sec^{-1})	k_{cat}/Km ($\mu M^{-1} sec^{-1}$)
BP	69 ± 10	432 ± 26	6,3 ± 1
BP-ChBD$_{CHIT1\text{-}72}$	98 ± 17	528 ± 49	5,4 ± 1,1
BP-ChBD$_{CHIT1\text{-}49}$	91 ± 6	343 ± 13	3,8 ± 0,3
P451A	92 ± 14	494 ± 39	5,4 ± 0,9
T452A	109 ± 16	359 ± 32	3,3 ± 0,6
G453A	93 ± 15	335 ± 28	3,6 ± 0,7
L454A	94 ± 17	293 ± 28	3,1 ± 0,6
V455A	89 ± 16	352 ± 32	4 ± 0,8
S457A	86 ± 19	305 ± 34	3,5 ± 0,9
N458A	120 ± 30	285 ± 39	2,4 ± 0,7
S459A	92 ± 17	386 ± 37	4,2 ± 0,9
K461A	73 ± 12	392 ± 32	5,4 ± 1
T464A	128 ± 33	368 ± 54	2,9 ± 0,9
W465A	78 ± 10	259 ± 16	3,3 ± 0,5
N466A	99 ± 16	348 ± 29	3,5 ± 0,6
